# Supplementary material for: Hippocampal Neurogenesis Is Enhanced in Adult Tau Deficient Mice
Source: Cells. 2020 Jan 14;9(1):210. doi: 10.3390/cells9010210 (PMC7016791; doi:10.3390/cells9010210)
Supplement: Supplementary file 1 [file cells-09-00210-s001.pdf]

Supplemental Data

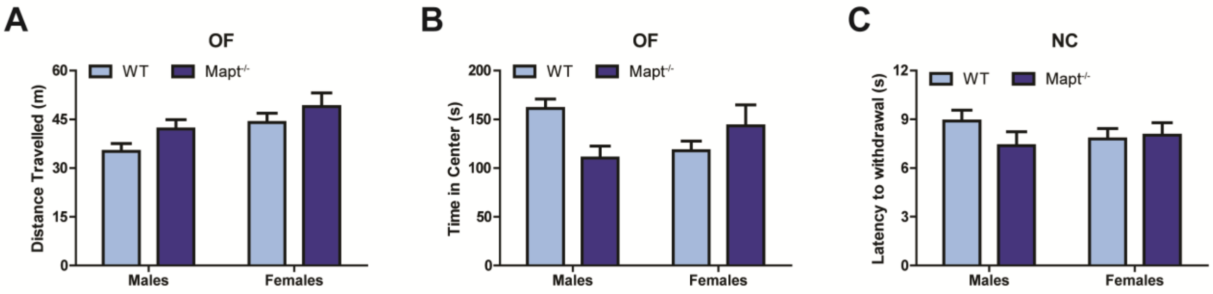

Figure S1. Anxiety and locomotion are not affected by sex in tau deficient mice. Data from Figure 1 broken down by sex for *Mapt*$^{-/-}$ and WT mice in the (**A-B**) the open field task and (**C**) hot-plate nociception. Data are represented as standard error of the mean (SEM) and analyzed by two-way ANOVA. WT, wild-type; OF, Open field; NC, nociception.

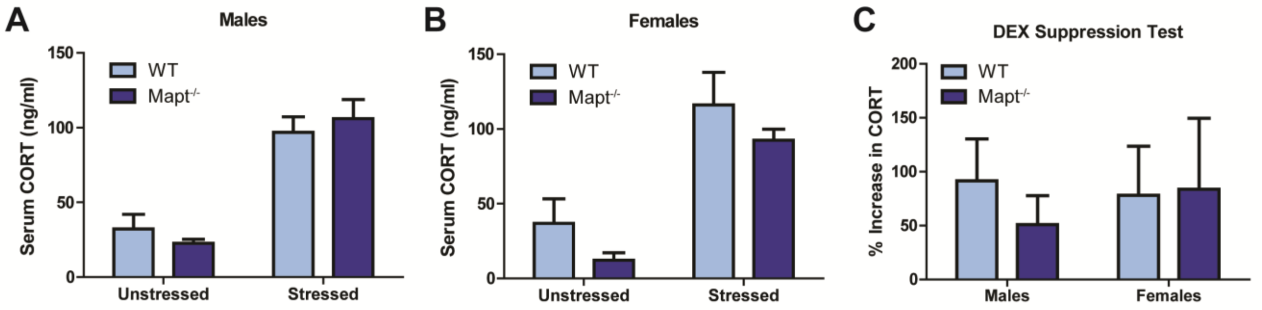

Figure S2. The HPA axis is not affected by tau deletion. Data from Figure 3 separated by sex for (**A**) basal and stressed serum corticosterone levels (ng/mL) as well as (**B**) CORT levels in response to dexamethasone. CORT, corticosterone; WT, wild-type.

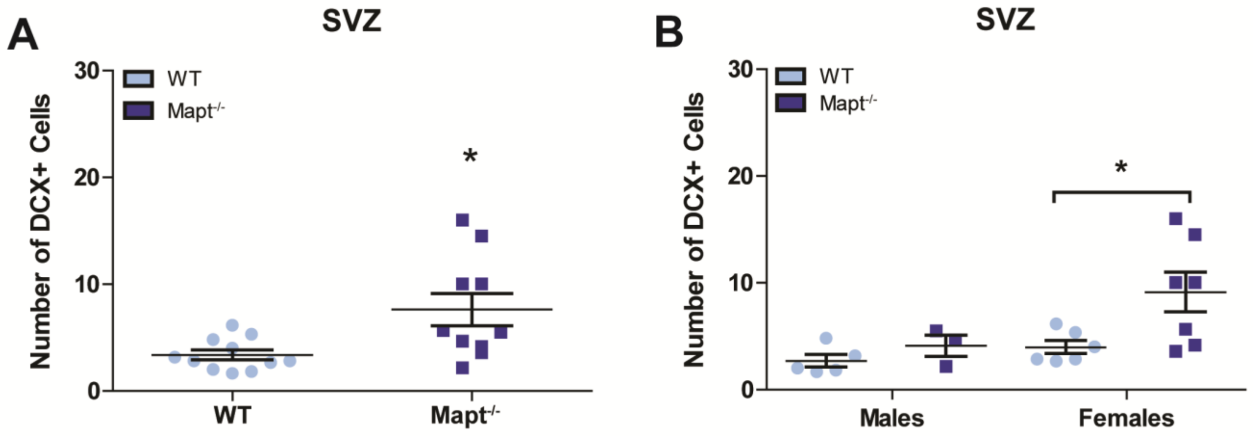

Figure S3. Neurogenesis is upregulated in *Mapt$^{-/-}$* mice. Doublecortin was measured by immunofluorescence in the SVZ subregion in brain tissue from *Mapt$^{-/-}$* and WT mice. (**A**) Quantification of DCX+ cells in the SVZ of the hippocampus is shown and also displayed by (**B**) sex. DCX+, doublecortin positive cells; SVZ, subventricular zone of the hippocampus; WT, wild-type.

Table 1. Top proteins associated to neurotigenesis in the anterior cortex and amygala.		
**Anterior cortex**		
**ID**	**Name**	**Expression Fold Change**
Rhob	Ras homolog family member b	4.706
Atcay	ATCAY kinesin light chain interacting caytaxin	2.388
Flna	Filamin A	2.326
Slc9a6	Solute carrier family 9 member A6	2.153
Ppp1r9a	Protein phosphatase 1 regulatory subunit 9A	1.97
App	Amyloid beta precursor	1.762
Cdk5	Cyclin dependent kinase 5	-1.453
Snap91	Synaptosome associated protein 91	-1.51
Gabrb3	Gamma-aminobutyric acid receptor subunit beta-3	-1.582
Gpm6a	Glycoprotein M6A	-1.832
Cdc42	Cell division cycle 42	-1.971
**Amygdala**		
**ID**	**Name**	**Expression Fold Change**
Gja1	Gap junction protein alpha 1	2.168
Ube3a	Ubiquitin protein ligase E3A	1.875
Strn	Striatin	1.787
Rapgef4	Rap guanine nucleotide exchange factor 4	1.769
Caprin1	Cell cycle associated protein 1	1.737
Lamb2	Laminin subunit beta 2	-3.257
Gap43	Growth associated protein 43	-3.275
Basp1	Brain abundant membrane attached signal protein 1	-3.444
Rims1	Regulated synaptic membrane exocitosis 1	-4.667
Fnbp1	Formin binding protein 1	-4.733

Table S1. Neural tissue homogenates from the amygdala (AMYG) and frontal cortex (FCX) of 14-month-old Mapt-/- and WT mice (n = 3 per genotype) were analyzed by proteomic mass spectrometry. Data includes top proteins associated to neuritogenesis in these brain regions.
